# Approaches, Strategies and Procedures for Identifying Anti-Inflammatory Drug Lead Molecules from Natural Products

**DOI:** 10.3390/ph17030283

**Published:** 2024-02-22

**Authors:** Tenzin Jamtsho, Karma Yeshi, Matthew J. Perry, Alex Loukas, Phurpa Wangchuk

**Affiliations:** 1 College of Public Health, Medical, and Veterinary Sciences (CPHMVS), Cairns Campus, James Cook University, Cairns, QLD 4878, Australia; karma.yeshi@my.jcu.edu.au (K.Y.); matthew.perry1@jcu.edu.au (M.J.P.); 2 Australian Institute of Tropical Health, and Medicine (AITHM), Cairns Campus, James Cook University, Cairns, QLD 4878, Australia; alex.loukas@jcu.edu.au

**Keywords:** natural products, biodiscovery approaches and strategies, anti-inflammatory drug lead molecules, isolation techniques, medicinal plants, parasitic helminths

## Abstract

Natural products (NPs) have played a vital role in human survival for millennia, particularly for their medicinal properties. Many traditional medicine practices continue to utilise crude plants and animal products for treating various diseases, including inflammation. In contrast, contemporary medicine focuses more on isolating drug-lead compounds from NPs to develop new and better treatment drugs for treating inflammatory disorders such as inflammatory bowel diseases. There is an ongoing search for new drug leads as there is still no cure for many inflammatory conditions. Various approaches and technologies are used in drug discoveries from NPs. This review comprehensively focuses on anti-inflammatory small molecules and describes the key strategies in identifying, extracting, fractionating and isolating small-molecule drug leads. This review also discusses the (i) most used approaches and recently available techniques, including artificial intelligence (AI), (ii) machine learning, and computational approaches in drug discovery; (iii) provides various animal models and cell lines used in in-vitro and in-vivo assessment of the anti-inflammatory potential of NPs.

## 1. Introduction

Over centuries, natural products (NPs) have played a crucial role in drug discovery, contributing significantly to the development of drugs and inspiring various strategies to identify novel drug candidates to treat diseases. Traditional medical systems, in particular, have presented insights into therapeutic effects, enhanced by the remarkable technological progress that has enabled incredible discoveries in natural product-based drug development. Similarly, bioinformatics and artificial intelligence (AI) have streamlined and improved the research and development processes associated with NPs [1].

New research methodologies and clinical, pharmacological, and chemical studies have resulted in devising better approaches to extraction, fractionation, and isolation of bioactive molecules, thereby facilitating drug discovery from NPs [2,3,4]. A notable example is the synthesis of the anti-inflammatory agent acetylsalicylic acid (aspirin), which is derived from the natural compound salicin (isolated from the bark of the willow tree, *Salix alba* L.), which revolutionised pain management for the masses [5].

Inflammation, derived from the Latin word “*inflammation*”, is a biological response activated by disruptions to tissue structures from various stimuli, which is commonly indicated as acute or chronic, depending on the response nature and resolution ability [6]. Acute inflammation, a short-lived response, involves vascular events, endothelial cell activation, leukocyte recruitment, and the release of substances from phagocytic cells [7]. Chronic inflammation persists due to unresolved acute responses, sustained inflammatory stimuli, persistent leukocyte migration, and the production of pro-inflammatory substances causing tissue damage and remodelling [8]. Chronic inflammation is now widely recognised for its involvement in conditions such as asthma, cancer, cardiovascular diseases, inflammatory bowel diseases (IBDs), neurodegenerative diseases, and rheumatic conditions. Understanding inflammation’s role in these diseases is critical for discovering and developing medicinal NPs for effective treatment [9].

Ulcerative colitis and Crohn’s disease represent the most common IBDs and manifest through symptoms including abdominal pain, diarrhoea, weight loss and damaged intestinal mucosa as a result of increased reactive oxygen species (ROS) production [10]. The pivotal pro-inflammatory cytokine in IBD is tumour necrosis factor (TNF), primarily produced by activated macrophages, monocytes, and T cells. Elevated TNF levels induce intestinal inflammation, impair tight junction function, cause apoptosis of epithelial cells, and heighten barrier permeability. This, in turn, triggers a cascade of pro-inflammatory cytokines such as interleukin-1 (IL-1) and interleukin-6 (IL-6), further exacerbating inflammation [9,11]. A systematic comprehension of the intricate mechanisms that underlie IBD is essential for determining treatment targets and, consequently, designing innovative drugs from natural products (NP). The utilisation of various murine models of IBD has significantly enhanced the understanding of IBD pathogenesis and has played a pivotal role in the formulation of novel therapeutic drugs [12].

Historically, indigenous NPs, primarily crude plant extracts/whole plant extract, were traditionally used by local healers/practitioners in treating inflammation-related disorders. For example, numerous plant species such as *Curcuma longa* L., *Plantago ovata* Phil., *Aloe vera* (L.) Burm.f., *Cannabis sativa* L., and *Artemisia absinthium* L. are popular remedies for treating IBD [12]. Similarly, a study revealed that among the 1881 new drugs approved from 1981 to 2019, 71 were unaltered NPs, 14 were botanical drugs, 356 were NP derivatives, and 424 were mimics of NPs [13]. In 2019, 9 out of 38 drugs approved by the FDA were derived from NPs [14]. 

The strategies for drug discovery from NPs evolve across various stages, with ancient knowledge inspiring modern drug discovery [15]. The isolation of active compounds, starting with morphine in 1806, marked the chemical primary stage. Prominent NPs, like quinine, caffeine, and nicotine, were isolated from NPs [16]. As NP isolation techniques improved, structural identification became a new direction in drug discovery, introduced by Robert Burns Woodward in the 1940s, significantly advancing research on compound structures [17]. Further, with the application of AI, more potential targets and increased compound libraries have been generated, thereby heightening new phases in drug discovery from natural products [18]. Furthermore, AI has demonstrated a crucial role in identifying potential hit and lead compounds, hastening the validation of drug targets, and optimising drug structure design, which has resulted in establishing a comprehensive framework for innovative approaches in drug discovery from NPs [19].

Several reviews have been written on NPs as sources of new drugs [20,21,22,23,24,25,26,27,28,29,30], yet no in-depth discussions of biodiscovery approaches and NP isolation and characterisation techniques have been reported previously. Hence, the current review aims to discuss the approaches used in selecting the starting materials, various extraction methods and modern techniques currently available for identifying anti-inflammatory small molecules from NP. Additionally, various murine models currently employed to validate the anti-inflammatory properties of compounds, elucidating both their potential and limitations, are also discussed herein. 

Overall, the literature search was conducted using terms (like natural product approaches, strategies, techniques, small molecules, anti-inflammatory, in vitro models, animal models, isolation and structure elucidation, and spectroscopic methods) in multiple databases, including Scopus, Web of Science, MEDLINE Ovid, PubMed, Google Scholar, and journal platforms. The information was compiled in a table and then analysed for approaches, techniques, and advances made in the anti-inflammatory methods. 

## 2. Approaches and Techniques for the Biodiscovery of Anti-Inflammatory Drug Leads

### 2.1. Approaches

Typically, the biodiscovery process involving NPs starts with identifying and selecting a suitable study species, such as a plant, animal, or microorganism. Strategic selection of raw materials is crucial for ensuring a high success rate of obtaining the targeted drug leads [31]. For the strategic selection of raw materials, three different approaches are commonly applied, namely (i) biorational approach, (ii) chemo-rational approach, and (iii) random or find-and-grind approach [15,32,33,34].

#### 2.1.1. Biorational Approach

The biorational approach uses the information derived from biological specimens that are used either in traditional medicines or have ecological functions in an organism/plant to develop drugs. This approach includes two search strategies: (a) Ethnobotanically/ethnopharmacologically directed strategy and (b) ecologically directed strategy [34]. 

##### Ethnopharmacology Approach

This is a field of study derives therapeutic agents from plants and animals traditionally used by people to treat various diseases [32,35]. Ethnopharmacology is considered a straightforward approach to drug discovery as it uses indigenous knowledge for the targeted isolation of drug leads [36]. Different cultures have invented their own ways of healing systems, and they exist mainly in two forms–scholarly traditional medical system (selected examples are Indian Ayurvedic medicine, Chinese traditional medicine, and Bhutanese Sowa Rigpa medicine) and oral/folklore medical system (selected examples are Australian aboriginal medicine, Kenyan medicine) [37]. Both medical systems contain enormous ethnopharmacological and ethnobotanical information that is resourceful for drug discovery and development [38]. Intuitively, the success rate of drug discovery using ethnopharmacological data is expected to be greater than with the random approach [36]. This approach has resulted in isolating several bioactive compounds, including anti-inflammatory SMs [39,40,41,42]. For example, triptolide was isolated from *Tripterygium wilfordii* Hook.f., which is traditionally used in TCM (Traditional Chinese medicine) to treat inflammatory-related diseases such as dermatomyositis, rheumatoid arthritis and systemic lupus erythematosus [43]. Triptolide inhibits iNOS gene expression by reducing the DNA binding activity of NF-κB and SAPK (stress-activated protein kinase) pathways [24]. Another example is the discovery of curcumin from *C. longa* by Vogel and Pelletierin in 1815 [44]. Curcumin had demonstrated anti-inflammatory activity by suppressing the activity of the NF-κB. Additionally, it showed inhibition of IL-1 and TNF, crucial cytokines in regulating inflammatory responses [45]. Other examples include apigenin (isolated from *Mentha australis* R.Br.), salicin (from *Salix alba* L.) [46,47], and protopine and kaempferol (from *Meconopsis* species of Bhutan) [38].

##### The Ecological Approach

This search strategy is driven by the chemical interaction of plants or organisms with their environmental factors, facilitated by SM, and is also called chemical ecology [48]. The secondary metabolites produced by organisms in response to interaction with their surroundings have mainly defensive roles (e.g., insect repellant or insecticidal properties of plants), and most of these defensive metabolites are reported to have diverse bioactive chemical constituents. Thus, this knowledge is often used to select starting materials not associated with ethnopharmacological knowledge in drug discovery [49]. The ecological approach has proven to be a compelling tool in drug discovery, particularly for determining habitats where drugs like anti-inflammatories could potentially be synthesised. Diverse influences, including habitat type, environmental stresses, and interactions between plants, microorganisms and hosts, affect this approach. These ultimately contribute to creating distinctive and various secondary SM with ecological functions, often including defence mechanisms [35,50]. According to Coley and Kursar [51], young leaves of the plant lack toughness and require unique chemical defence, which may not be needed once leaves mature. Considering this concept, Colery et al. conducted a comparative study on young and mature leaves of 18 Panamanian woody species to incorporate this ecological concept in selecting the starting materials for drug discovery [51]. Following thin-layer chromatography and Dragendorff’s reagent test, 24 different alkaloids were detected, of which 71% were found in young leaves and 29% in mature leaves [52]. Another example includes a study conducted to assess the variation in terpenes and phenolics during leaf development across 81 tropical species, which observed that levels of total phenols and proanthocyanidins were nearly double in young leaves compared to mature leaves [2]. Furthermore, monoterpenes, sesquiterpenes, and diterpenes were higher in young leaves than their mature leaves [3,4]. Recent studies conducted by Wangchuk’s group at James Cook identified the pharmaceutical potential of Australian Wet Tropics plants affected by climate change, and they have also discovered two novel drug lead molecules from one of the ecologically-directed tropical montane cloud forest plants [53,54]. Similarly, potent small molecules anti-inflammatory excretory-secretory products (ESP) derived from parasitic helminths were identified using metabolomics and lipidomics platforms [55,56].

Similar comparative studies on salt marsh plants and marine worms indicate that chemical defences are more robust in tropical populations than in temperate ones [52]. For example, SM fucoxanthin, obtained from brown algae, exhibits the ability to suppress the expression of cyclooxygenase 2 (COX-2) protein and downregulate the production of prostaglandin E2 (PGE2). Similarly, pheophytin, derived from *Enteromorpha prolifera* (O.F.Muller) J.Agardh, effectively inhibits superoxide radical and inflammatory responses induced by 12-O-tetradecanoylphorbol-13-acetate in murine macrophages [57]. This suggests that regions closer to the equator experience higher environmental stress, leading to the synthesis of unique bioactive secondary metabolites [58]. Hence, the ecological approach accelerates the identification of potential drug-lead compounds from NP. 

#### 2.1.2. Chemorational Approach

The chemorational approach in drug discovery is grounded on chemotaxonomic information, including cheminformatics of a botanical specimen and targeted phytochemical class assessments [34]. In this approach, preliminary evaluation of chemical classes and examining families and genera known for their analogous compounds with prior biological activities are necessary to increase the chance of getting the targeted drug lead [59]. Studies suggest that species belonging to the same genus or closely related genera tend to yield compounds with similar chemical structures. For instance, the chemical classes isoquinoline and indole alkaloids were reported to be produced by 164 genera belonging to 47 families [60]. Similarly, seven distinct plant species belonging to the Solanaceae family synthesise the same tropane alkaloid hyoscyamine [59]. Structural information from the scaffolds of the existing drugs could also form a basis for driving drug lead screening and discovery. The existence of this information in advance would speed up the screening timeframe and achieve better bioactivity results. However, this approach limits screening to what is already known, potentially missing out on the discovery of new scaffolds from unstudied NP sources [34].

#### 2.1.3. Random Approach/Find-and-Grind Approach 

This approach involves collecting samples randomly and screening them for any biological hits. The selected NPs, following a random approach, are usually screened for the desired chemical classes, including flavonoids, terpenoids, phenols, steroids, and alkaloids, and their bioactivities, if any [61]. This approach is often known as “find and grind”, which involves randomly selecting NPs without prior ethnopharmacological, ecological or chemical information [32,34]. It primarily involves random high-throughput screening (HTS) of crude extracts for phytochemical and biological activity. Based on biological hits, the best samples are selected for the further isolation and characterisation of drug leads. The success rate of this approach in generating drug leads is comparatively lower than it is for the other two approaches. For instance, the National Cancer Institute (NCI) in the United States randomly screened 35,000 plant species in search of anti-cancer agents and discovered only two drug leads, paclitaxel and camptothecin [61].

### 2.2. Recent Advances in Anti-Inflammatory Drug Discovery Approaches

#### 2.2.1. Computer-Aided Drug Discovery

Despite technological advancements, high attrition rates still exist in target selection, hit identification, lead optimisation, and clinical candidate selection. This has extended the drug discovery and development duration to an average of 10–15 years with an approximate cost of $2 billion [62]. The initial phases of discovery and preclinical efforts contribute to over 43% of expenses [63]. To address this challenge, the integration of extremely effective and time-efficient strategies, such as computer-aided drug discovery [64], when merged with traditional drug development techniques, has significantly boosted the probability of drug discovery from NPs [65]. 

The computer-aided drug discovery [64] approach has demonstrated a high success rate in screening large compound libraries to identify expected active compounds for experimental testing, lead compound optimisation to enhance affinity and drug metabolism and pharmacokinetics (DMPK) properties, including absorption, distribution, metabolism, excretion, and potential toxicity (ADMET) and designing novel compounds [66]. This approach is broadly classified as structure-based drug design (SBDD) and ligand-based drug design (LBDD). In principle, SBDD integrates target and ligand, including techniques such as ligand docking, ligand design, and pharmacophore modelling, while LBDD methods predict activity relying on the information of likeness/difference of the known active ligand/inactive [67].

SBDD methods include molecular dynamics, molecular docking and structure-based virtual screening that enhance binding site interactions and ligand affinity in drug discovery [68]. On the contrary, LBDD methods are applicable for unknown target structures that use 2-dimensional (2D) or 3-dimensional (3D) structures of known ligands interacting with the target. However, modern approaches often integrate both LBDD and SBDD techniques for a more comprehensive strategy. Quantitative structure-activity relationship (QSAR) is commonly used in predicting biological activity from chemical structures [67]. For instance, in a study conducted by Geronikaki et al. [69], a CADD approach employing PASS (Prediction of Activity Spectra for Substances) analysis and sample size software was utilised to predict the biological activity of 573 in silico-designed compounds, specifically thiazole/benzothiazoles, and benzoisothiazoles. Among the compounds, 31 were predicted to have anti-inflammatory properties, and 22 were identified as non-specific COX and specific 5-LOX (lipoxygenase) inhibitors. A total of nine compounds were selected for in-vitro assays, of which eight showed anti-inflammatory activity in carrageenin-induced paw oedema. Of all, 2-(thiazole-2-ylamino)-5-(m-chlorophenylidene)-4-thiazolidinone for COX-1 and COX-2, and 2-(thiazole-2-ylamino)-5-(m-nitrophenylidene)-4-thiazolidinone for 15-LOX were proposed as the most active compounds based on docking studies [69]. A similar study was conducted by Liaras et al. [70] identified thiazole and thiazolidinone derivatives as promising COX and LOX inhibitors. 

#### 2.2.2. Artificial Intelligence (AI) in Drug Discovery

Machine and deep learning methods are now widely used in drug discovery, including virtual screening, toxicity prediction, structure-activity relationship, pharmacophore modelling, and physiochemical activity [71]. The AI advancements have offered a promising opportunity to enhance drug design rationale [72]. Molecular modelling, virtual screening, exploration of NP libraries, and database mining are a few examples of such advancements employed to enhance the outcomes in drug discovery [73]. Computational-based analysis, such as in silico simulations, predicting drug binding sites or targets through structure-activity relationships has increased the likelihood of finding promising lead compounds [74]. For example, the Bayesian machine learning algorithm, BANDIT, incorporates multiple data types and has reportedly predicted around 4000 target compounds with an impressive 90% accuracy rate [75]. An alternative approach involves the application of pharmacophore-based in silico simulations to appraise the physicochemical characteristics of interactions between targets and ligands, relying on the 3D model of the pharmacophore [50]. Recently, AI has been used in NMR spectrum analysis, phase and baseline corrections, which is crucial, especially for high-density spectra like proton spectra. Traditional methods face challenges with such dense signals, prompting the application of deep learning for improved results. Bruker’s scientists introduced a deep learning-based approach in TopSpin software 4.1.3, excelling in phase and baseline correction for 1D 1H NMR spectra and achieving human-level accuracy. They also developed a deep learning algorithm for automated signal region detection, showcasing the potential for fully automatic extraction and analysis of information in NMR spectra, advancing towards comprehensive automation [76].

Pharmaceutical companies grappling with rising research and development costs adopt rules like Lipinski’s and drug-likeness filters [77]. In this view, machine learning models have exhibited a significant role in forecasting absorption, distribution, metabolism, and excretion (ADME) by linking these properties to molecular characteristics and establishing intricate structure-property relationships across various molecular structures and mechanisms [78,79,80]. For example, XenoSite’s neural network achieves 87% accuracy in predicting metabolism sites, while deep learning (DL) methods have been utilised to automatically handle a variety of chemical features, ensuring robust predictions in toxicity [81]. Furthermore, AI-driven tools such as DP4-AI and MS2DeepScore can predict and identify the metabolites through a clustering analysis [82,83]. However, “bioactivity hits” identified through HTS screening may not consistently present strong activity when assessed in vitro. To respond to this challenge, researchers may explore phenotypic cell assays in a semi-HTS mode, utilising extracts from NPs [84]. The phenotypic screen-based approach seeks to identify a promising lead compound before identifying a specific target and the subsequent optimisation of the lead. In contrast, the molecular target screen-based approach involves pre-determined target identification before embarking on HTS screening [74,84].

### 2.3. Methods for Extracting, Purifying, and Determining the Structures of Anti-Inflammatory SMs from NPs

#### 2.3.1. Extraction and Fractionation 

Extraction is the first step in the NP isolation process, which differs slightly depending on the extraction materials. For marine NPs like fresh seaweeds, initial cleaning with tap water is followed by washing seawater to remove sand particles, epiphytes, and other impurities. Subsequently, the cleaned seaweeds were rinsed with distilled water before preparing extracts [85]. For metabolomic studies, the extraction procedure is different. For example, the somatic tissue of the parasite is chilled with phosphate-buffered saline and centrifuged, and the supernatant is discarded. The samples are further freeze-thaw and centrifuged. The supernatant is used for metabolomic and lipidomic analyses [86].

Most of the time, washed samples are dried before extraction. For drying, either cryogenic, oven, open-air or freeze drying is used based on the experimental aims and raw materials. NP materials like ESP/parasites need freeze drying, while terrestrial and aquatic plants prefer cryogenic, oven, or air drying [85,87]. However, to extract oil from the plant materials, air drying by maintaining a moderate temperature with proper ventilation is essential to preserve the extracted oil’s flavour, aroma, and therapeutic properties [87]. On the other hand, dried plant samples are ground into coarse powder for extraction. Precautions must be taken during extraction to prevent compound degradation and the development of artefacts [22]. 

Selecting a suitable solvent system is crucial and hinges upon the raw material, target compounds, and final product, with consideration for solute-solvent polarity matching and stability during extraction [61,73,88]. Non-polar solvents like hexane, chloroform, dichloromethane, and ethyl acetate and polar solvents like water, acetone, methanol, and ethanol are commonly employed [89]. Each solvent’s unique properties suit specific polarities; for instance, ethanol–water mixtures are recommended for phenolic extraction, while acetone has proven effective in polyphenol extraction compared to methanol, water, and ethanol [90]. Overall, extraction efficiency is influenced by various factors, including the types of solvent used, the powder size of the raw material, solvent-to-solid ratio, extraction temperature and the extraction duration [91,92]. For hydrophilic compounds, water, methanol, ethanol, acetone and ethyl acetate are used as solvents, while dichloromethane or dichloromethane/methanol (1:1) is used for the extraction of lipophilic compounds [93,94,95]. In a study conducted to examine the phytochemical constitute of grape pomace, six different solvents, including 80% methanol, 80% ethanol, ethyl acetate, acetone, 50% and 80% methanol (acidified) were used as a solvent for extraction. The suitable solvent was optimised based on varying phytochemical polarities, which revealed that ethyl acetate was the most efficient solvent for the extraction of polyphenols-compounds, 50% methanol (acidified) for anthocyanin isolation, and acetone for extracting ursolic acid [96]. Hexane is often used to remove chlorophyll during extraction from leaves. 

Traditional extraction, such as reflux extraction, percolation, maceration and soxhlet extraction, commonly uses large amounts of organic solvents and demands extended extraction duration [88]. Recent extraction methods, including microwave-assisted extraction, solid-phase extraction, supercritical fluid extraction, micro-extraction, surfactant-mediated methods, and pressurized-liquid extraction, have been employed for extraction from the natural products, which offer advantages such as reduced solvent consumption and sample degradation, negating extra clean-up and pre-chromatographic steps, enhanced extraction efficiency, selectivity and kinetics [23,97].

Thermolabile compounds have been successfully extracted using the maceration method. One example is the polyphenolic compound catechin isolated from the fruits of *Arbutus unedo* L. following the maceration approach, which showed the same extraction yields as that of the recent approach (ultrasound and microwave-assisted extraction techniques) [98]. The efficiency of maceration depends on varying aspects such as saturation time, percolating solvent, solid-liquid ratio, and particle size of raw materials [99]. At the same time, maceration is a simple yet time-consuming method with a relatively low yield. Hence, combining maceration with modern approaches such as microwave and ultrasonic assistance has significantly enhanced extraction efficiency, as demonstrated by yielding a larger quantity of volatile compounds in the citrus peel extraction [100].

Percolation is a continuous extraction method performed at room temperature and is suitable for heat-sensitive substances. On the other hand, the percolation approach displays drawbacks, including substantial solvent usage and time-consuming extraction [101]. However, varying concentrations of ethanol are used as solvents to prevent solvent volatile loss. In general, factors such as powder size, solvent composition, extraction time, percolation flow rate, and solvent amount determine the efficacy of extraction [102,103]. In a study on phenolic extraction from *Allium sativum* L., percolation exhibited higher extraction and recovery rates than maceration [104]. Similarly, comparing volatile component extraction from grapeseed oil, percolation yielded 60 components, whereas Soxhlet extraction produced 67 [105]. 

The reflux extraction method employs volatile organic solvents to enhance extraction rates and reduce solvent usage. However, it is unsuitable for thermolabile raw materials due to prolonged heating [103]. The study has shown that reflux extraction is less efficient than modern methods like ultrasound and microwave-assisted extraction [106]. For instance, studies on apigenin extraction from *Scutellaria barbata* D.Don demonstrated that ultrasound-assisted extraction yields higher and faster results than reflux extraction [107]. Soxhlet extraction, the continuous reflux extraction method, provides improved extraction efficiency and requires less solvent than reflux extraction, addressing its limitations [103]. This method is commonly employed for the extraction of phenolic compounds and oils. For example, a study on the extraction of phenolic compounds from *Vernonia cinerea* (L.) Less. leaves using the Soxhlet method demonstrated higher yields [108]. However, Soxhlet extractors may generate unwanted by-products due to the heat needed for extraction, affecting heat-sensitive components [109]. 

In contrast to conventional extraction techniques, ultrasonic and microwave-assisted extraction methods are recognised as more environmentally friendly and economically feasible approaches for obtaining NPs. These methods substantially decrease extraction time, improve efficiency, and simultaneously minimise solvent consumption. They serve as energy-efficient and emission-reducing alternatives to traditional extraction methods [110,111]. For example, the use of ultrasonic and microwave-assisted extraction methods resulted in higher yields of phenolic compounds from olive leaves and polysaccharides from *Camptotheca acuminata* Decne. fruits, respectively [112,113]. Supercritical fluid extraction, a sustainable technology, employs carbon dioxide for its favourable properties like moderate critical pressure and temperature, non-toxicity, and environmental friendliness. This method surpasses traditional methods, offering shorter extraction times and increased yield rates [114]. The increased yield rates, as evidenced by essential oil extractions from camphor trees, are one such example [115]. Pressurised liquid extraction is considered eco-friendly and recognised for low solvent usage, speed, high recovery, and reproducibility [116]. Comparative studies, like polyphenol extraction from pomegranate peels, affirm its efficacy [117]. 

#### 2.3.2. Phytochemical Screening 

The study often asserts that the medicinal use of NP mixtures proves more effective than purified compounds, attributing this efficacy to beneficial “synergistic” interactions. However, the mechanisms underlying these synergistic effects in NP largely remain unknown [118]. Understanding the nature of synergistic activity within NP extracts is crucial for optimising safe and efficacious disease treatments [119]. Conversely, research findings have discussed antagonism, where the effects of active constituents are obscured by other compounds in an NP mixture [119]. To address this safety concern and to enhance the effectiveness of NP mixtures, it is essential to thoroughly characterise bioactive mixtures, including determining the concentrations of the phytochemical constituents and isolating and identifying the small molecules that contribute to their biological activity.

Natural product-derived chemicals are broadly classified as (i) phenolic compounds (flavonols, anthocyanins, flavanones, isoflavonoids, flavones, and catechins), (ii) terpenoids (glycosides, carotenoids, sterols, and saponins) and (iii) alkaloids (cyanogenic glycosides and glucosinolates) [120,121]. These phytochemical classes have demonstrated various biological activities [122]. For example, the *Dodonaea viscosa* Jacq. leaf extract, abundant in terpenes, demonstrated anti-inflammatory effects by reducing carrageenin-induced rat paw oedema [123]. Considering this finding, Hautriwaic acid was isolated from the same plant species that displayed anti-inflammatory activity by reducing inflammation in 12-O-tetradecanoylphorbol 13-acetate induced mice ear edema [124].

**Figure 1:**
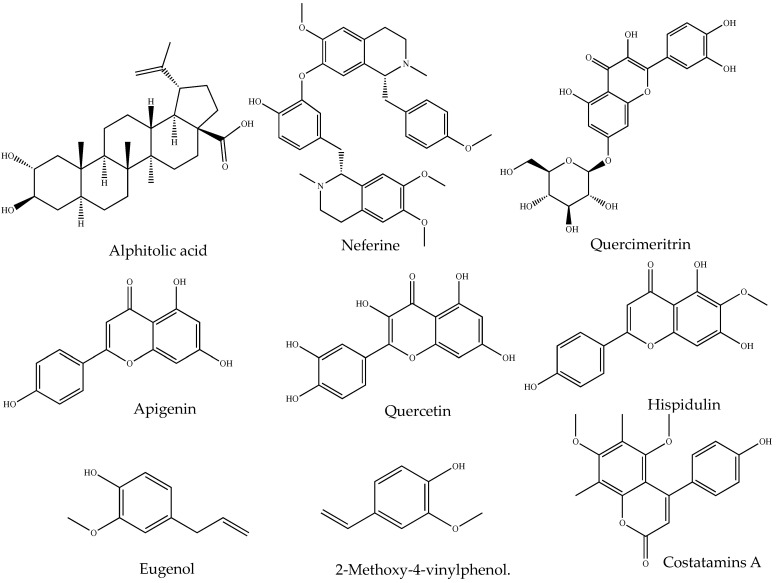
Chemical structures of representative anti-inflammatory small molecules from natural products.

Flavonoids are well known for their anti-inflammatory properties, which are demonstrated by their inhibition of inflammation-related regulatory enzymes and transcription factors [125]. For instance, *Ageratum conyzoides* L. leaf extract, rich in flavonoids, has shown anti-inflammatory effects in subacute and chronic inflammation in rats [126]. This guided the isolation of 5′-methoxy nobiletin and Eupalestin (flavones), which reduced p65 NF-κB (nuclear factor kappa B) and p38 MAPK (mitogen-activated protein kinase) activities [127]. Similarly, studies have reported the anti-inflammatory potential of alkaloids [128], coumarins, and glycosides in both in vitro and in vivo experiments. Therefore, evaluating major phytochemical classes in biological samples offers insights into potential biological activities. This is achieved by mixing the crude extract of samples with different test reagents that produce colour changes, indicating the presence or absence of phytochemical classes [129]. Hence, the qualitative screening of those SM may aid in the process of extracting, fractionating, purifying and identifying bioactive compounds for human use [130]. The examples of phytochemical class and associated isolated anti-inflammatory small molecules are given in Table 1, and the representative structure is in Figure 1. Table 2 shows different test methods for identifying major classes of phytochemicals. 

#### 2.3.3. Isolation of Anti-Inflammatory SMs (Bioactivity-Guided) 

Isolation of bioactive compounds from crude extracts involves repeated fractionation, followed by testing of fractions for biological activity (Figure 1). The success in isolating the pure compounds from NP is reinforced by implementing bioassay-guided separation techniques [91]. Repeated fractionation and testing are required in this approach, which is expensive as it consumes large amounts of solvents and reagents [147]. Non-bioassay-guided purification is cost-effective, but there are risks of losing bioactive agents during purification, particularly those minor compounds. Thus, selecting a suitable method is crucial to obtain maximum target compounds [148].

**Figure 2:**
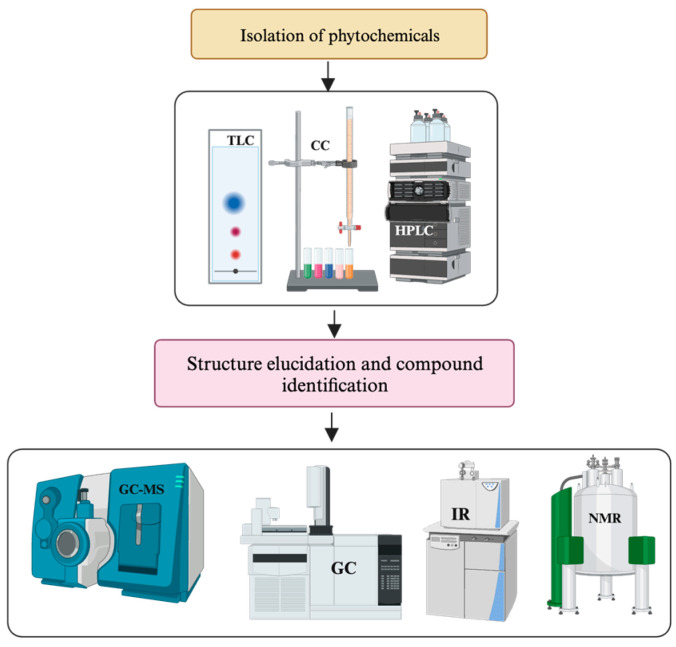
General framework for identifying SMs and determining the structure of new phytochemicals.

In most cases, chromatographic techniques such as thin-layer chromatography (TLC), column chromatography (CC), liquid chromatography (LC), gas chromatography (GC) and high-performance liquid chromatography (HPLC) are recommended for the fractionation and purification of compounds [149,150,151]. However, none of these methods alone offers a complete solution to overcome purification and isolation challenges. Ideally, the best result is typically achieved by combining different techniques [152]. For instance, the preliminary fractionation of crude extracts of NPs using organic solvents, followed by successive separation through CC and ultimately isolating compounds using HPLC or PTLC, have exhibited greater success in getting pure compounds [153]. Additionally, the chromatographic profiles of TLC, CC and HPTLC assist in identifying suitable mobile phases, thereby enhancing the purification and separation efficiency of the compounds from NPs [23,97,154,155] (Figure 2). 

##### Thin Layer Chromatography/Preparative TLC

TLC operates on the principles of adsorption chromatography, established upon differences in solute interactions with a thin adsorbent layer fixed onto substrates such as plastic, aluminium, or glass plates [156]. The compounds in the mixture will move to different positions on the plate according to their solubility, and each spot representing a separated compound can be identified by comparing the retention factor (rf) of the TLC profile with that of known compounds [97]. The spotted compounds are collected at different locations and re-extracted using various solvents [157]. Combining TLC with mass spectrometry (MS) through compound extraction has enhanced the spectral data available for selected compounds [158,159]. For instance, phenolic compounds such as rutin and chlorogenic acid could be identified and quantified using a TLC-MS extraction interface coupled with NMR [160]. Preparative thin-layer chromatography (prep-TLC) is helpful in purifying compounds on a smaller scale if the sample is less than 100 mg [161]. For example, in a recent study by Albayrak et al., a total of eight compounds, including two novel coumarin glycosides, 7-methoxy isoarnottinin 4′-O-rutinoside, and 7-methoxy isoarnottinin 4′-O-β-D-glucopyranoside were successfully isolated from the root extract of *Prangos heyniae* H.Duman & M.F.Watson using this chromatographic technique [162]. 

##### Column Chromatography

Column chromatography isolates organic compounds from NP and achieves the desired outcomes using various mechanisms such as ion exchange, molecular sieve, and adsorption chromatography [91]. Vacuum liquid chromatography (VLC) is commonly chosen for fractionating crude extracts and isolation due to its simplicity and high sample capacity [163]. As evident, VLC approaches, CC and prep-TLC have isolated three new compounds (anthraquinones, naphthalene, and naphthoquinones) and five known compounds from Asphodeline lutea [163]. In another example, repeated use of VLC led to the isolation of spinasterol, an antimutagen, from *Cucurbita maxima* Lam. flowers. Like VLC, low-pressure liquid chromatography (LPLC) is also a significant tool in fractionating and isolating natural products as the only separation steps or in tandem with other chromatographic techniques. A new compound, wasabolide, exhibiting anti-neuroinflammatory activity, was isolated from the roots of *Wasabia japonica* (Miq.) Matsum. employing the LPLC approach along with TLC and HPLC [164]. Considering the nature of the compound targeted to purify and isolate from NPs, either normal phase or reverse phase chromatography techniques are used [165]. Normal phase chromatography employs a stationary polar phase (typically silica gel) and a nonpolar mobile phase. Compounds are separated based on their polarities, with more polar compounds interacting more strongly with the stationary phase and eluting later [165,166]. Isolation of anti-inflammatory compound 2,4,6-trihydroxybenzo-phenone-4-O-geranyl ether from *Hypericum sampsonii* Hance is one such example [167]. On the contrary, reverse phase chromatography uses a nonpolar stationary phase (usually a hydrophobic alkyl chain-bonded silica) and a polar mobile phase. Due to its reproducibility, most high-performance liquid chromatography (HPLC) separation strategies predominantly optimize the classical reversed-phase liquid chromatography (RPLC) approach [168]. However, the RPLC/RPLC combination, characterised by similar separation principles, demonstrates limited orthogonality. To address this limitation, researchers have incorporated hydrophilic interaction chromatography (HILIC) to purify polar compounds from complex samples or provide complementary selectivity alongside the RPLC approach [169]. Within this context, integrating RPLC and HILIC methodologies is an effective strategy for efficiently isolating bioactive compounds from diverse natural products. Compounds are separated based on their hydrophobicity, with more hydrophobic compounds being retained in the stationary phase for longer isolation [165,166]. For example, the anti-inflammatory compound tunicoside B was isolated from *Dianthus superbus* L. via reversed-phase liquid chromatography [169].

Flash chromatography (FC) is another valuable chromatographic technique primarily used to fractionate crude extract rapidly. The commonly used stationary phase of FC includes silica gel with a particle size of ca. 40 μm, ensuring better resolution and separation of compounds [160]. This method, commonly referred to as medium-pressure liquid chromatography, offers a swift approach to isolating and purifying compounds compared to traditional column chromatography. Applying monitored medium pressure to the column facilitates the separation of compounds in significant sample quantities, resulting in a superior quality of purified compounds [170]. To this line, myristicin, 3′-hydroxy- and 3′-methoxypuerarin, puerarin, and daidzin were isolated from the crude extract of *Pueraria lobata* (Willd.) Ohwi. by combined centrifugal partition (CPC) and FC methods [171]. The current advancements include fully automated flash chromatography equipment equipped with robotic fraction collectors and online detection units, significantly enhancing the efficiency of separating, isolating, and purifying constituent compounds within a complex mixture of crude extract and identification [172].

##### High-Pressure Liquid Chromatography (HPLC)

HPLC is commonly used for the purification and isolation of SM compounds from NPs due to its high sensitivity and specificity [173]. Biologically active compounds are often found in NP extracts as a minor constituent. HPLC offers the ideal resolving power for efficiently handling such multi-component samples in analytical and preparative contexts [174]. Typically, the detection and isolation of phytochemicals via HPLC can be achieved using an isocratic system. However, multiple samples with different retention factors are analysed using a gradient elution method [175]. To achieve optimal separation, various parameters, including mobile phase, flow rate, appropriate detectors and the right columns, need to be optimised [97]. For example, hydrophobic compounds exhibit longer retention time (rt) due to a strong affinity for the nonpolar stationary phase, while hydrophilic molecules have shorter rt in a polar stationary phase [176]. Overall, the right mobile phase can be determined based on the polarity of the solvent. Aside from identifying and isolating unknown compounds from natural products, HPLC methods are crucial in de-replication, that is, identifying known metabolites in extracts ideally at an initial phase of the fractionation process [177,178,179]. For instance, the anti-inflammatory SM emodin was isolated from *Rumex dentatus* L. following gradient elution in HPLCs [180]. 

##### Ultra-Performance Liquid Chromatography (UPLC)

Ultra-Performance Liquid Chromatography (UPLC) has emerged as the next evolutionary stage above HPLC methodologies. UPLC consists of particles with diameters less than 2 µm in the stationary phase, and employing short columns facilitates increased pressures, ultimately producing narrower liquid chromatography peaks. Beyond offering enhanced chromatographic separations with narrow peaks, UPLC significantly reduces analysis times, often to 10 min or less [181].

The reduced size of UPLC particles shortens the diffusion path, enhancing efficiency and yielding 2–3 times higher sensitivity in detection compared to HPLC. Advances in instrumentation and column technology have been instrumental in achieving remarkable increases in UPLC’s speed, resolution, and sensitivity, making it a driving force in the contemporary pharmaceutical industry, particularly in conjunction with mass spectrometry [182,183]. 

A notable advantage of UPLC is its seamless conversion of HPLC methodology, maintaining identical conditions such as temperature and eluents. UPLC results differ significantly from HPLC, showcasing increased resolution, enhanced throughput, reduced analysis time, decreased solvent usage, lower solvent disposal, and an overall cost reduction, including up to an 80% reduction in solvent usage [184,185]. For example, to study the metabolic profiling of *Ammi majus* Walter Roots, ultra-high-performance liquid chromatography coupled with mass spectrometry (UPLC/MS-MS) was selected. The study revealed that coumarins such as Xanthotoxin and (iso) arnottinin (the most abundant) and coumarins including bergaptol-O-hexoside, dihydrochalcone (phloretin), coumestrol, and bergaptol were reported. Conversely, the negative acquisition mode revealed the presence of flavonoids and phenolics, with *p*-coumaroyl tartaric acid and 3,7-dimethylquercetin as the most abundant [186].

#### 2.3.4. Identification of SMs and Structure Elucidation of Novel Molecules

**Figure 3:**
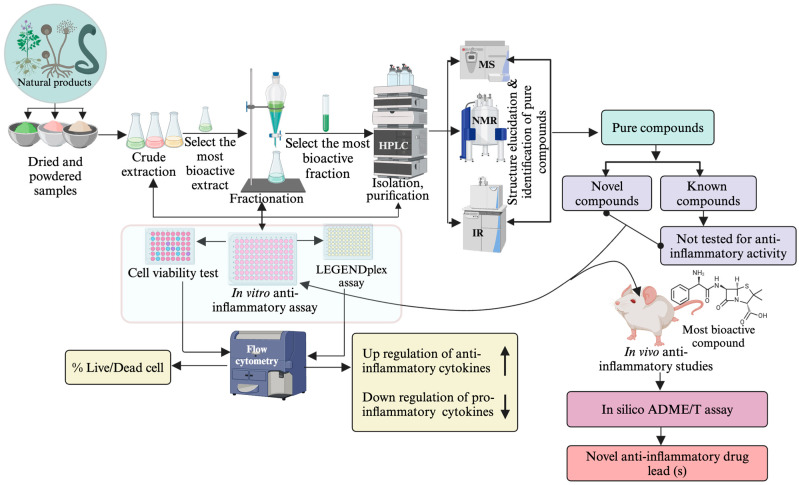
Bioassay-guided anti-inflammatory drug-lead identification approach.

Different spectroscopic instruments such as LC-MS, UV, IR, NMR and HRMS are used to obtain spectroscopic information from purified compounds [187]. While LC-MS/MS gives mass, HRMS provides molecular formula, IR gives functional group information, and NMR gives 1D and 2D spectral data, which are used to determine the structure with the help of software [155]. Overall, applying these spectroscopic techniques enhances pure compound extraction, fractionation, isolation and structural elucidation [20,188,189]. A general framework for determining the structure of new phytochemical compounds is outlined in Figure 3. These advanced instruments are also used in metabolomics analysis of crude plant extracts, which can identify known compounds and predict novel SMs.

##### Nuclear Magnetic Resonance (NMR) Spectroscopy

Most NMR systems today function on a 300 to 1020 MHz frequency [190] and operate by showcasing the differences in magnetic resonance of selected spin-half nuclei within a molecule. The ^1^H and ^13^C nuclei are by far the most frequently studied in isolated compounds, though other nuclei such as ^15^N, ^19^F and ^31^P, among many more, can be studied to provide additional information. In 1D NMR spectroscopy, all nuclei of a given type are excited with a specific resonance frequency, providing information about the chemical environment and some limited information about the positional arrangement of atoms within a molecule [187,191,192]. 

To gather more structural information about the exact configuration and stereochemistry of the different nuclei in a molecule, 2D NMR experiments are employed. By utilising different pulse sequences, correlations between nearby nuclei can be observed, providing detailed information regarding the connectivity between atoms within a molecule. The gradient correlation spectroscopy (gCOSY) experiment shows 2–4 bond correlations between ^1^H-^1^H nuclei, while the gradient total correlation spectroscopy (gTOCSY) displays these interactions without a bond limit within each spin system [155]. The gradient nuclear overhauser effect spectroscopy (gNOESY) experiment shows through-space ^1^H-^1^H correlations, providing information regarding the physical structure in space. Heteronuclear techniques such as the gradient heteronuclear single quantum coherence (gHSQC) and gradient heteronuclear multiple bond correlation (gHMBC) provide 1-bond and 2–4 bond correlations between ^1^H and ^13^C nuclei, respectively [34,193]. All of these experiments are typically run on each sample to provide a detailed map of correlations between nuclei to enable the effective elucidation of novel compounds [34,193].

##### Liquid Chromatography-Mass Spectrometry (LC-MS/LC-MS/MS)

LC-MS involves the separation of mixtures using HPLC and subsequent measurement of the mass using mass spectrometry [176]. MS operates by ionising a compound, which is then separated and identified based on their mass-to-charge ratio (*m*/*z*). Additionally, LC-MS demonstrates a short time frame of analysis and is widely used in determining, identifying and quantifying drug metabolites from natural products [194]. The technique also demonstrates high resolving power and accuracy and reveals the analytes in complex samples, even at low concentrations [195]. For instance, in a recent study by Wang et al. [196], 8 out of 31 compounds from *Cullen corylifolium* (L.) Medik. were identified by LC-MS techniques. Another notable advantage of LC-MS includes the ability to determine known and unknown SM from NPs [197]. Compound identification relies on the structural information deduced from the fragmentation pattern of the molecular species, which is generated through collision-induced dissociation [198] or collision-activated dissociation (CAD) in MS experiments. This approach enhances the ability to avoid repetitive isolation of known and unknown compounds (dereplication) and enables targeted isolation of compounds, thereby minimising time and resources to identify lead compounds [199]. However, the information obtained from a single LC-MS analysis may prove insufficient for confirming the structures of certain molecules. The introduction of tandem mass spectrometry (MS–MS) has addressed this issue. Consequently, the hybridised LC-MS–MS technique has proven highly valuable and essential for the analysis of natural products [200]. For example, LC-MS/MS approach revealed the presence of 102 compounds in *Hericium erinaceus* (Bull.) Persoon, including organic acids (31), nucleotides and analogues (10), amino acids [201], carbohydrates and derivatives (6), flavonoids (5), unsaturated fatty acids (3), terpenoids (3), phenolic acids (3), phenylpropanoid (1), steroid (1), other compounds (32) [202].

##### UV-Visible Spectroscopy

UV spectroscopy, described as highly sensitive with a significant level of detectability, reveals UV-absorbing chromophores in a molecule. Thus, UV-visible spectroscopy serves for qualitative analysis and identification of certain compound classes in pure and biological mixtures [192]. It is beneficial for quantitative study due to the strong chromophoric nature of aromatic molecules in the UV range. UV-visible spectroscopy is applied to detect phenolic compounds, such as anthocyanins, tannins, polymer dyes, and phenols, which form iron complexes detected by UV-Visible spectroscopy [187]. Despite being less selective, spectroscopic UV-Visible techniques provide insights into the composition of total polyphenol content. For example, studies have utilised UV-Visible spectroscopy to determine total anthocyanins, phenolic acid, and flavones at 520, 360, 280 and 320 nm, respectively. In this view, UV-visible spectroscopy demonstrates a time-efficient and cost-effective alternative to other techniques [203].

##### Infrared Spectroscopy (IR)

Infrared spectroscopy (IR) has been a foundation in chemistry since the 1900s and is used to identify common functional groups based on specific peaks in the spectrum [204,205]. IR spectroscopy stands out for its speed, affordability, and non-destructive nature, reducing or eliminating sample preparation time [206]. It is highly sensitive, requiring only a small sample amount, and accommodates a wide range of matrix types—solids, powders, films, gels, liquids, and gases—without generating waste [207]. However, challenges arise in interpreting spectra from intricate mixtures and the necessity to establish and maintain resilient calibration models for quantitative analysis [208]. Further, the need for an exhaustive database and the complexity and overlapping nature of spectral features limit its automated structure elucidation potential. For example, identifying particular functional groups like the carbonyl peak at approximately 1700 cm^−1^ is straightforward; however, unravelling the fingerprint region (400–1500 cm^−1^) poses a more challenging endeavour [209]. 

Generally, the IR spectrum has two main regions: the functional group region (4000–1200 cm^−1^) and the fingerprint region (1200–400 cm^−1^). Most functional groups exhibit absorption in the former, while the latter is unique to the compound. For instance, 2-pentanol and 3-pentanol, despite similar functional group absorption, differ in their fingerprint regions sample [210]. Accurate identification involves comparing the compound’s fingerprint area with that of a known sample [210]. While advanced methods like NMR and LC-MS outshine IR spectroscopy in structure elucidation, they have drawbacks, such as cost and time requirements. IR spectroscopy remains valuable for its speed, affordability, non-destructiveness, and user-friendliness [209].

##### High-Resolution Mass Spectrometry (HRMS)

High-Resolution Mass Spectrometry (HRMS) instruments, either operating independently or coupled with separation techniques, have significantly advanced the characterisation of plant secondary metabolites, making HRMS-based methods the preferred choice for structural elucidation and quantification. For instance, a study on anti-inflammatory phytochemicals in Plantago major extract, utilising HRMS coupled with UHPLC, identified various compounds, including Ostruthin, erucamide, *cis*-7-hexadecanoic acid, oleic acid, palmitoleic acid, inoleic acid, ethyl palmitoleate, conjugated linoleic acid, *trans*-3-indole acrylic acid, hexadecanamide, palmitic acid, methyl palmitate, oleamide, and 4-oxododecanedioic acid [211]. In metabolomics, HRMS is highly beneficial for computing elemental compositions and determining isotopic ratios. HRMS instruments offer resolutions ranging from 10,000 to several million, showcasing their versatility. The profiling of *Micromelum falcatum* Tanaka extracts using HPLC-DAD (diode array detector), and UPLC-ESI^+^-HRMS is a valuable approach for targeted coumarin isolation and dereplication. Employing a dereplication strategy, 7-oxygenated coumarins were detected, with eight coumarins identified and three identified as new natural products: microfalcrin, microcoumaririn, and micromelosidester. HRMS and HRMS/MS analysis revealed specific patterns for the rapid detection and characterisation of 7-methoxylated coumarin derivatives [212]. However, challenges include the high cost, maintenance demands, and specialised training needed for HRMS instruments, limiting their feasibility in standard labs. Analysing large data blocks requires specific software and expertise, and identifying unknown compounds is hindered by reference database limitations and structural elucidation challenges [213,214].

## 3. Screening Isolated Compounds for Anti-Inflammatory Properties

### 3.1. In Vitro Assays for Anti-Inflammatory Screening of SMs and Crude Extracts

SMs sourced from NPs offer the potential bioactive drug lead compounds. In this view, a rapid and cheap method or high throughput system (HTS) to assess crude extracts and purified compounds for their anti-inflammatory properties is required to speed up the drug discovery process [215]. There are a number of bioassays to identify anti-inflammatory extracts and SMs, and some of the commonly used techniques are briefly discussed here.

#### 3.1.1. Human Leukemia Monocytic Cell Line (THP-1)

THP-1 monocytes have been extensively used to study monocyte, macrophage, and dendritic cell functions, mechanisms, signalling pathways and nutrient and drug transport. THP-1 monocytes can readily be differentiated into M1 or M2 macrophages or dendritic cells in the laboratory through the addition of defined cytokine and growth factor cocktails. This cell line is widely used to assess the modulation of monocyte, macrophage and dendritic cell activities. This cell line exhibits a differentiation pattern resembling primary monocytes and macrophages, with the homogeneous genetic setting contributing to their high reproducibility and low phenotypic cellular variability [216]. Further, THP-1 cells can be maintained in vitro for up to 3 months without losing sensitivity or activity. It has been described as a model for immunological regulation [217,218]. 

The THP-1 cell line is currently used in IBD research due to its ability to mimic inflammatory responses accurately [55,219,220,221]. For example, Xue et al. investigated the anti-inflammatory activities of a crude extract of *Vaccinium macrocarpon* Aiton in LPS-stimulated human THP-1 monocytes, which resulted in significant downregulation of IL-6 and TNF at concentrations of 0.1–10 μg/mL compared to LPS control [222]. A similar study using THP-1 macrophages showed that curcumin and chlorogenic acid (CGA) synergistically down-regulated production of the pro-inflammatory cytokine TNF and COX-2 enzyme [223]. In another study, 4-methylguaiacol significantly inhibited the secretion of various inflammatory cytokines and inflammasome components (TNF, IL-1β, IL-6, IL-8, NO, and PGE_2_) by LPS-activated THP-1 cells through the nuclear factor erythroid 2-related factor 2 (Nrf2) signalling pathway and blocking the NF-κB and AP-1 signalling pathways [224]. At large, the THP-1 cell line is a widely accepted model for screening for anti-inflammatory activities from crude extracts or compounds prior to undertaking in vivo studies [225,226].

#### 3.1.2. Caco-2 Cell Line

Caco-2 is an immortalised colon cancer cell line derived from human colorectal adenocarcinoma cells [227]. The differentiated Caco-2 cells exhibit features that closely resemble small bowel enterocytes, including brush-border microvilli [228,229], and produce various cytokines, like IL-6, IL-8, IL-15 and TNF [230,231,232]. As such, Caco-2 cells are widely used in IBD research [233,234]. Zhang et al. [235] investigated in vitro anti-inflammatory effects of *Paenibacillus polymyxa* Prazmowski exopolysaccharide (PYQ1-EPS) on TNF-induced inflammation in Caco-2 cells. At a dose of 300 mg/L, PYQ1-EPS blocked the stimulation of mitogen-activated protein kinase (MAPK) and NF-κB signalling pathways, inhibited pro-inflammatory cytokines (IL-1β, IL-6, IL-8, and IL-12A), and enhanced the production of the anti-inflammatory cytokine IL-10, thereby suppressing inflammation [235]. Hence, Caco-2 cells serve as a tool for examining and confirming the ethnopharmacological applications of plant extracts for treating inflammatory-related disorders such as IBD. For example, a study conducted to examine the anti-inflammatory properties of two classes of flavonoid compounds, namely, polymethoxylated flavones and prenylflavonoids revealed that IL-1β-stimulated Caco-2 cells exhibited a significant anti-inflammatory response [236]. 

#### 3.1.3. Human Colorectal Adenocarcinoma Cell Line (HT29)

HT29 cells derived from a colon adenocarcinoma represent the epithelial lining of the intestine and, when stimulated, mimic the inflammatory conditions linked to IBD. Consequently, HT29 cells serve as valuable in vitro models, enabling the exploration of cellular and molecular mechanisms underlying IBD pathogenesis and assessing potential interventions [237]. Many studies have used this cell line to evaluate the anti-inflammatory properties of NPs. *Alpinia officinarum* Hance hexane extract (HEAO) downregulated the expression of *NF-κB* and *COX-2* genes in HT29 cells [238]. Jo et al. investigated α-onocerin, lyclavanin, 24-*O*-acetyl-serratenediol, lycoclavanol, and lyclaninol, which down-regulated IL-8 in LPS-stimulated HT-29 cells [239]. 

In another study, the anti-apoptotic effect of curcumin was investigated on IFNγ-stimulated HT-29 cells, and curcumin treatment significantly reduced IL-7 production, possibly by downregulating the NF-κB signalling pathway [240]. Another study by Kim et al., using the HT-29 cell line, investigated the impact of luteolin on the production of the chemoattractant cytokine IL-8. Luteolin reduced IL-8 levels in HT-29 cells induced by TNF, and moreover, it inhibited phosphorylation of p38 mitogen-activated protein kinase (p38MAPK), prevented inhibitor of κB (IκB) degradation, and inhibited nuclear factor-κB (NF-κB) activation [241]. 

#### 3.1.4. The murine Macrophage Cell Line (RAW 264.7)

RAW 264.7 is a macrophage-like Abelson leukaemia virus-transformed cell line derived from BALB/c mice and has been widely employed to study intestinal inflammation, including IBD [242,243,244,245]. This cell line can precisely replicate the conditions of the gut environment and effectively mimic Toll-like receptors (TLRs), which play an essential role in the development of IBD [246,247,248,249]. RAW 264.7 cells exhibit an inflammatory response to inflammatory stimuli like LPS and determine their potential impact on primary cells [250,251]. For example, compounds that reduced the expression of pro-inflammatory cytokines or NO in RAW264.7 cells stimulated by LPS are often viewed as likely anti-inflammatory candidates for human use [252]. Another example is 3-cinnamoyltribuloside isolated from *Camellia nitidissima* C.W.Chi., which downregulated TNF-α, IL-1β, and IL-6 in LPS-stimulated RAW 264.7 cells [253]. Treatment of LPS-stimulated RAW 264.7 cells with methanol extract of *Amandine* species downregulated expression of proinflammatory cytokines, including IL-6, TNF, iNOS, and COX-2 [247]. Although RAW 246.7 cells are commonly used in various studies, it is worth noting that RAW cells, which are not cloned, may undergo changes in their phenotype (surface marker and expression of macrophage characteristic gene) and function (NO production) over time in continuous culture (passage no. > 30) [254,255]. However, following the standard of 100 ng/mL of LPS, cell densities ranging from 1 to 9.9 × 10^5^ cells/well and incubation periods of 12 to 24 h in an atmosphere of 5% CO_2_ at 37 °C are recommended as the optimum condition [255]. 

#### 3.1.5. Peripheral Blood Mononuclear Cells (PBMCs) Assay

Human PBMCs consist of dendritic cells, lymphocytes (T-cells, B-cells and natural killer (NK) cells), and monocytes, which produce cytokines as markers on stimulation by activators [256,257]. The cytokine profile variations and changes in activation marker expression, particularly by T cells, could indicate whether the immune response is Th1-, Th2-, Th17-, or regulatory T cells (Treg)-dependent response [257]. Hence, the PBMC stimulation assay is widely used for assessing anti-inflammatory activity in IBD research [258,259,260]. Paprocka et al. investigated the anti-inflammatory activity of five natural SMs using LPS- LPS-stimulated PBMC, which showed that all derivatives significantly inhibited the production of pro-inflammatory cytokines, TNF and IFN-γ [261]. In another study, IPX-18 suppressed the production of IFN-γ, TNF, IL-8 and IL-2, with an IC_50_ value of 96.29, 103.7, 122.9 and 105.2 nM by PBMC induced with 50 ng/mL of phorbol-12-myristate-13-acetate (PMA) [262]. In the IBD clinical setting, TNF increases effector T cells at the site of inflammation, while IFN-γ and IL-2 stimulate immune cells, and IL-8 recruits neutrophils and other immune cells to the site of inflammation [263,264,265]. Hence, the ability of IPX-18 to reduce the production of pro-inflammatory cytokines signifies its anti-inflammatory potency [262]. Despite being widely used for anti-inflammatory screening, the PBMC assay has shown limitations or phenotypic differences between immune cells and those in the intestinal mucosa [237,257]. For example, the immune cells of the intestinal mucosa consist of natural killer T cells (NKT cells), innate lymphoid cells (ILCs), and B lymphocyte cells (B cells), which are absent in blood cells [257]. These differences suggest that PBMC isolated from blood will exhibit different responses compared to the mononuclear cells of lamina propria [257]. The advantages and limitations of different cell lines used in IBD are briefly discussed in Table 3. 

### 3.2. In-Vivo Experimental Models for Anti-Inflammatory Screening

The complex aetiology of inflammatory diseases such as IBD poses challenges in delineating their respective pathophysiologies [269]. IBD complexity arises from the interplay between genetic, immunological, and environmental factors and intestinal microbiota, and a comprehensive understanding remains elusive. In this setting, animal models are indispensable tools for establishing a more refined preclinical environment to target specific elements involved in IBD pathogenesis [269,270]. Further, animal models in IBD research have proven to be crucial tools for addressing scientific queries, appraising preclinical efficacy, probing pharmacokinetics and piloting safety assessments [271]. Diverse animal models for IBD have been established to screen and identify new anti-inflammatory drug leads [270]. Approximately 66 IBD animal models, including transgenic mouse, bacteria-induced, genetically engineered, mutation knock-in, chemical-induced, spontaneous, and adoptive transfer models are available. However, none of these models can encompass all facets of intestinal inflammation mechanisms [272,273]. Among animal models, chemical colitis (e.g., TNBS-induced and DSS-induced colitis), spontaneous *Winnie* mice and T-cell transfer models have been widely used in IBD drug discovery [274].

#### 3.2.1. 2 2,4,6-Trinitrobenzene Sulfonic Acid (TNBS)-Induced Colitis Model

TNBS is a short acute model of IBD. It is a haptenising molecule with no inherent antigenicity, yet its administration elicits a Th1-mediated immune reaction, including the infiltration of CD4^+^ T-cells, neutrophils and macrophages, resulting in transmural colitis mirroring CD in humans [275]. For example, lamina propria CD4^+^ T cells obtained from mice treated with TNBS exhibited increased secretion of the Th1 cytokine IFN-γ, mirroring the cytokine profile observed in lamina propria CD4^+^ T cells isolated from patients with CD [276]. Commonly used mouse strains include SJL/J, BALB/C and C57BL/6. Intrarectal administration of 0.5 mg of TNBS in 50% ethanol to mice induces colitis with notable symptoms, including rectal prolapse, weight loss and diarrhoea, mirroring specific attributes of CD witnessed in humans [277,278]. In a similar trend, TNBS induces significant changes in the morphological features and pharmacological responsiveness of the muscular layer in the distal colon when compared to the proximal colon in mice, closely mirroring features of human UC [279]. Besides its application in investigating the underlying causes of Crohn’s disease, TNBS colitis serves as a cost-effective and valid method for evaluating the potential therapeutic benefits of traditional medicines and natural compounds [277]. For instance, the compound berberine [280], reportedly used in traditional Chinese medicines in treating inflammatory-related diseases [281], exhibited a protective effect in mice with TNBS-induced colitis through inhibition of proinflammatory cytokines, including IFN-γ, IL-17, IL-6, IL-1β, and TNF [282]. While it has some advantages, including a short and fast model, technical ease and cost-effectiveness, the reproducibility of the results from TNBS experiments remains challenging. 

#### 3.2.2. Dextran Sodium Sulfate (DSS)-Induced Colitis Model

DSS is a water-soluble compound commonly used in IBD models, and its ability to induce colitis depends on several factors, including age, sex, strain of the mice, dosage concentration and mode of administration [283]. This animal model uses DSS to induce ulcerative colitis in mice and replicates histopathological characteristics resembling those observed in clinical practice among human ulcerative colitis (UC) [283,284,285]. Further, oral administration of DSS induces colitis that is mostly confined to the colon [286] and initiates upregulation of various chemokines, cytokines, NO and iNOS concentrations [287,288]. For example, DSS administration initiates an immune response by releasing IL-1β, activating ILC3 and upregulating IL-23, where IL-23 initiates recruitment of neutrophils and CD4^+^ T cells and amplified IL-17 signalling, causing chronic inflammation, characterised by depletion of goblet cells and heightened commensal microbiota adhesion [275]. Likewise, DSS induces progressive and severe colitis, enhancing NF-κB activation [289].

C57BL/6 mice with a Th1 genetic background develop severe, chronic DSS-induced colitis, which becomes aggravated over time with ascending doses, whereas BALB/c, with a Th2 genetic background, recover from acute disease upon dose reduction [290]. Overall, the changes in physiological and cytokine profiles observed in the DSS-induced IBD model serve as valuable indicators for investigating IBD’s underlying causes and assessing the impacts of potential therapeutic interventions.

#### 3.2.3. Oxazolone ((4-Ethoxylmethylene-2-Phenyloxazol-5-One)-Induced Colitis Model (OC))

Oxazolone is a classical haptenising agent extensively employed to induce colitis in mice that can facilitate the assessment of the underlying pathological mechanisms contributing to the progression of UC. Acute oxazolone colitis (OC) is induced by a one-time application of oxazolone enema. In contrast, chronic OC is induced by pre-sensitization through dermal exposure five days prior to the enema [291,292]. However, in both UC and CD, mucous membrane inflammation, epithelial microulcerations, infiltration of neutrophils, macrophages, lymphocyte cells, decline in the goblet and epithelial cells with the appearance of bleeding, and vascular dilation present the histological traits that resemble human UC [275,293,294,295,296]. This colitis model elicits an immune response by releasing IL-25, activating ILC2, and producing IL-13, which activates CD4^+^ T cell responses, intensifying the type-2 cytokine production [275]. Various mouse strains have been utilised to observe immune responses. The C57BL/6J (C57/BL6 or C57/BL10) and SJL/J strains primarily exhibit Th1-mediated immune reactions, whereas BALB/c manifests a pronounced inclination toward a Th2 phenotype resistant response [297].

#### 3.2.4. Winnie Mouse Model of Colitis

Most of the animal models of IBD are associated with acute colitis induced by chemicals such as DSS or TNBS [298]. With limited animal models that mirror the spontaneous chronic intestinal inflammation of humans, the *Winnie* mouse model of colitis derived from a C57BL/6 background has become an important animal model exhibiting the clinical indicators of IBD [299]. This model displays chronic intestinal inflammation due to a missense mutation in the Muc2 mucin gene and shows a reduced expression of the *Muc2* gene, which is also often seen in human UC and CD [300,301]. In a study to investigate the intestinal microbiome and metabolome’s association in the inception of IBD (treatment and recovery), *Winnie* mice have demonstrated more distinct microbial shifts and metabolic profile changes in their faeces that resemble human IBD [302]. Hence, the *Winnie* mouse model offers a valuable tool for studying chronic intestinal inflammation in IBD, given its similarity to human IBD manifestations. 

#### 3.2.5. T Cell Adoptive Transfer Model 

The T cell transfer model has been described as the exemplary and well-described chronic colitis model stimulated by interference of T cell homeostasis [303]. Among the various mouse models used to comprehend the origins of IBD, the CD4^+^ CD45RBhi T cell adoptive transfer model has been extensively utilised to investigate the initiation and control of immunopathology in chronic colitis driven by T cells [304]. Established in the early 1990s [305], the CD4^+^ CD45RB^high^ T cell adoptive transfer model has been categorised into CD45RB^high^ and CD45RB^low^ subtypes based on the expression levels of CD45RB that exhibit unique cytokine responses to various stimuli [306,307].

The investigation demonstrates that transferring naive CD4^+^ CD45RB^high^ T cells from healthy wild-type [308] mice into T and B cell-deficient syngeneic recipients leads to the development of pancolitis and small bowel inflammation within 5–8 weeks after the T cell transfer [64,309]. Similarly, Morrissey et al. revealed that transferring CD45RB^high^ CD4^+^ T cells from healthy mice to SCID (severe combined immunodeficiency disease) mice results in non-bloody diarrhoea, weight loss, and mortality within 4–8 weeks [305]. However, co-transferring CD4^+^ T cells inhibits colitis, indicating their crucial regulatory role [310]. Specifically, introducing WT CD4^+^ CD45RB^high^ T cells into RAG KO mice triggers persistent inflammation in both the small and large intestine, while transferring the same T cells into TCR KO mice elicits only mild duodenitis and colitis [311]. These findings demonstrated inflammation in small and large intestines, which may help study the disease pathogenesis and additional pathways of immune regulation [311]. Overall, the chronic nature of colitis in T-cell transfer models closely mirrors human IBD. These models offer flexibility, allowing researchers to select T cell types and recipient mice based on investigative goals. This versatility facilitates the study of specific T cell subsets’ roles in intestinal homeostasis and inflammation. The models provide high experimental control over treatment timing and other factors in colitis development. While T cell adoptive transfer models excel in investigating IBD mechanisms and testing potential therapies, limitations exist. These models do not precisely replicate the human immune system, and variations in intestinal microbiota between mice and humans may restrict their full representation of human IBD intricacies [303]. Table 4 presents the advantages and disadvantages of different animal models of IBD. 

## 4. Conclusions and Future Directions

Plant-derived natural compounds are a pivotal reservoir for novel pharmaceuticals targeting various human disorders, notably those associated with chronic inflammation. To optimise the extraction of therapeutic agents from these natural products (NPs), a myriad of systematic methodologies have been devised for selection, identification, isolation, characterisation, and biological screening. The efficacy of drug discovery from NPs hinges on search strategies such as ethnobotanical/ethnopharmacological approaches, ecological considerations, and random sampling. Ethnopharmacology, leveraging indigenous knowledge for targeted drug lead isolation, has demonstrated a commendable success rate. Conversely, while contributing to drug discovery, ecological and random approaches necessitate additional inputs, especially in the initial screening phase. 

Overall, the qualitative screening approach enables the identification of phytochemical classes, guides the extraction and identification of bioactive compounds, and evaluates major phytochemical classes in biological samples for potential therapeutic applications. Further, integrating artificial intelligence through computer-aided drug discovery has revolutionised the exploration of intricate phytoconstituent profiles. This has resulted in the isolation or synthesis of numerous therapeutic drugs and novel lead compounds, forming foundational scaffolds for future drug development. In this regard, a comprehensive and interdisciplinary approach, amalgamating traditional and ethnopharmacological knowledge with disciplines such as phytochemistry, botany, analytical chemistry, suitable biological screening strategies, and modern drug development tools, is imperative for yielding successful outcomes in this domain.

The isolation of natural products (NPs) is a meticulous process involving critical steps such as sample washing, selecting drying methods based on experimental goals, and implementing precautions to prevent compound degradation. Extraction efficiency is intricately linked to solvent choice, powder size, and duration. The selection of solvents is tailored to the characteristics of the compound, utilizing water, methanol, ethanol, acetone, ethyl acetate, or dichloromethane as needed. While traditional methods persist for thermolabile compounds, Soxhlet extraction provides convenience, albeit with potential by-product generation. Comparative analyses highlight the efficacy of refluxing for specific compounds, while recent advancements promise reduced solvent usage and heightened efficiency. Chromatographic techniques, including TLC, CC, VLC, HPLC and UHPLC, play crucial roles in extracting and purifying anti-inflammatory secondary metabolites, with spectroscopic methods like NMR, LC-MS, IR, and UV-visible spectroscopy aiding in structure identification and elucidation. Using in vitro assays allowed researchers to gain insights into the compounds’ potential anti-inflammatory effects before progressing to more complex in vivo studies. Selecting the best model for studying anti-inflammatory activity involves considering various factors. For example, PBMC assay is widely utilized in IBD research, assessing cytokine profiles and activation markers. However, limitations arise due to phenotypic differences between PBMCs and intestinal mucosal cells. Similarly, RAW 264.7 cell replicates gut conditions. Still, it requires careful consideration of continuous culture changes, as cells may change their phenotype (surface marker and expression of macrophage characteristic gene) and function (NO production) over time. Likewise, THP-1 is also used as it provides high reproducibility, accurately mimicking inflammatory responses in IBD. However, none of these in vitro assays embrace all facets of intestinal inflammation mechanisms; hence, the choice depends on specific research requirements. 

Similarly, selecting an appropriate in vivo model for testing anti-inflammatory activity hinges on research goals and desired model characteristics. The TNBS-induced colitis model offers fast, cost-effective results, mimicking crohn’s disease. The DSS-induced colitis model replicates human ulcerative colitis, providing insights into IBD’s underlying causes. The oxazolone-induced colitis model exhibits UC like histopathological traits, while the winnie mouse model reflects spontaneous chronic intestinal inflammation. The T Cell adoptive transfer model is a well-described chronic colitis model, allowing flexibility in studying specific T cell subsets. Therefore, it is important to use an interdisciplinary approach that incorporates traditional and ethnopharmacological knowledge and expertise in phytochemistry, botany, analytical chemistry, appropriate biological screening strategies, and modern drug development tools for the successful discovery of natural product-based drug leads. 

## Figures and Tables

**Table 1:** Examples of phytochemical classes and isolated small molecules showing anti-inflammatory activities.

Source	Class of Organic Compound	Isolated Small Molecules	Anti-Inflammatory Activity
*Angophora costata* (Gaertn.) Britten	Alkaloid	Costatamins A	Suppressed NO production and decreased TNF secretion in RAW 264.7 cells [131]
*Nelumbo nucifera* Gaertn.		Neferine	Decreased the production of IL-6 and TNF in RAW 264.7 cells activated by lipopolysaccharide activated [132]
*Ochrosia elliptica* Labill.		10-Methoxyconolidine	Decreased the level of TNF, IL-6 and NO production in lipopolysaccharide stimulated RAW 264.7 cells [133]
*Alphitonia petriei* Braid & C.T.White	Terpenoid	alphitolic acid	suppressed the production of NO and TNF in RAW 264.7 cells activated by lipopolysaccharide + IFN-γ [134]
*Centipeda minima* (L.) A.Braun & Asch.		Centiplide A	Suppressed the production of NO in RAW 264.7 cell treated with liposaccharides [135]
	Tannins	Tannic acid	HaCaT cells exposed to UVB irradiation when treated with tannic acid, inhibited production of the proinflammatory cytokine IL-18, IL-1, IL-6, TNF, COX-2, and PGE2 and elevate its mRNA expression [136]
*Clerodendrum inerme* Gaertn.	Flavonoid	Hispidulin	suppressed the production of PGE2 and expression of the expressions of iNOS and COX-2 by blocking NF-κB DNA-binding activity and JNK pathway [137]
*Nelumbo nucifera* Gaertn.		Quercetin	Reduced NO production in lipopolysaccharides treated RAW 264.7 cells [138]
*Merremia tridentata* (L.) Hallier f.		Apigenin	Inhibit IL-1β, IL-6 and TNF production in lipopolysaccharide induced murine BV2 microglia cells [139]
*Ipomoea pes-caprae* (L.) R.Br.	Phenolic	Eugenol and 2-Methoxy-4-vinylphenol.	Reduced the synthesis of prostaglandins [140].
*Barringtonia racemose* (L.) Spreng.	Glycoside	Barringoside I	Exhibited a moderate inhibition NO production in lipopolysaccharide stimulated RAW 264.7 cells [141]
*Brasenia schreberi* J.F.Gmel.		Quercimeritrin	Suppressed the expression iNOS and NO in lipopolysaccharide-stimulated RAW 264.7 cells [142]

NO: Nitric oxide; TNF: INF-γ: Interferon-gamma; TNF: Tumor necrosis factor; IL-1β: Interleukin-1 beta); IL-6: Interleukin-6; COX-2: cyclooxygenase-2; PGE2: prostaglandin E2; iNOS: inducible nitric oxide synthase; JNK: the c-Jun N-terminal Kinase.

**Table 2:** Types of tests for detecting major phytochemical classes in a crude extract [129,130,143,144,145,146].

Types of Tests	Reagent/Chemical Added to Extract	Confirmatory Color Change
Alkaloid test		
Dragendorff’s test	Potassium bismuth iodide solution (1 mL)	Orange, red precipitate
Wagner’s test	Potassium iodide solution (1 mL)	Reddish brown precipitate
Mayer’s test	Potassium mercuric iodide solution (1 mL)	Whitish or cream
Hager’s test	Saturated ferric solution (1 mL)	Yellow-colored precipitate
Steroid test		
Libermann Burchard’s test	Acetic anhydrites + sulfuric acid	Violet to blue-colored ring
Terpenoid test		
Copper acetate test	Copper acetate solution (3–4 drops)	Emerald, green color
Salkowski’s test	CHCL_3_ and concentrated H_2_SO_4_ (2 and 3 mL respectively)	Reddish brown color
Tannins test		
Gelatin’s test	Gelatin solution + sodium chloride (1%)	Appearance of white precipitate
Flavonoid test		
Lead acetate test	Lead acetate solution (2–3 drops)	Yellow precipitate
Alkaline reagent test	Sodium hydroxide solution (2–3 drops)	Initially yellow color and turns colorless after adding dilute acid
Phenolic test		
Ferric chloride test	Ferric chloride (2–3 drops)	Bluish-black color
Lead acetate test	Lead acetate (2–3 drops)	Yellow color
Gelatine test	Gelatin solution (1%)	White precipitate
Mayer’s reagent test (potassium mercuric iodide test)	Mayer’s reagent (1 mL)	white precipitate
Anthraquinone test		
Bontrager’s test	Boiled extract (In 10% of HCL for 2–3 min) Add CHCL_3_ to filtrate (2–3 drops of 10% NH_3_) Heat mixture (3–4 min)	Pink color

**Table 3:** Advantages and limitations of different cell lines for IBD.

Cell Line Assay for IBD	Advantages	Limitations
*THP-1 (Human leukemia monocytic cell line)*	Exhibits resemblances to human monocytes, including morphology, secretory products, oncogene expression, membrane antigen expression, and genes related to lipid metabolism [266]. Support in vivo-validated biological activity of compounds and the pharmacological effects of drugs [266]. THP-1 cells are relatively simple and safe for use, as there is no report of toxicity or presence of infectious viruses. THP-1 cell line can be cultivated in vitro for approximately three months, maintaining consistent cell sensitivity and activity. Following standard protocol, cells can be stored for several years and revived without impacting monocyte–macrophage characteristics and cell viability. Homogeneous genetic background reduces the phenotypic variability and facilitates reproducibility [225,267].	Cellular background and cultivation under controlled conditions may induce different sensitivities and responses compared to normal somatic cells in their natural environment [226]. Produce less amount of inflammation-related cytokines compare to PBMCs [216].
*Caco-2 cell line*	Display similar traits of small intestinal enterocytes, including transporters and enzymes essential for drug metabolism [216]. Ability produces various cytokines, including IL-6, IL-8, IL-15, TNF, and thymic stromal lymphopoietin (TSLP) [230]. Possess features like enterocytes in the small intestine (presence of brush-border microvilli) [256].	Lacks mucus layer which may impact the delivery and interaction of anti-inflammatory agents with the underlying cells [216]. The presence of other cell types besides enterocytes and several non-cellular elements poses challenges to the research [256].
*HT29 (Human colorectal adenocarcinoma cell line)*	Represent the epithelial lining of the intestine, mimicking inflammatory conditions associated with inflammatory bowel disease (IBD). Valuable in vitro model for studying the cellular and molecular mechanisms involved in IBD pathogenesis and evaluating potential interventions.	Considered as pluripotent intestinal cell line, as alterations in the culture media can induce various enterocytic differentiation pathways [216]. Extended differentiation duration and elevated glucose consumption [36] result in undifferentiated cells, even in the presence of high glucose concentrations [256].
*The murine macrophage cell line (RAW 264.7)*	Replicate the gut environment and effectively mimic Toll-like receptors (TLRs), imperative in the pathogenesis of IBD [249]. RAW 264.7 cells, derived from BALB/c mice, share similarities with monocytes/macrophages and demonstrate versatility, making them valuable for initial screening and minimizing the need for animal use [268]. Exhibits a strong and established inflammatory response, when challenged with lipopolysaccharide and Toll-like receptor 4 (TLR4) agonist [250,268].	Potential changes in RAW cells during continuous culture. May have to consider co-culture of RAW cells Caco-2 cells to study anti-inflammatory effects effectively [216].
*Peripheral blood mononuclear cells (PBMCs) assay*	Relevant model for studying anti-inflammatory responses as nit is human-derived. Easily isolated from peripheral blood, ensuring accessibility for experiments. Enable functional assays, measuring cytokine production, proliferation, and cytotoxicity to gain insights into immune responses. Valuable for drug screening, facilitating the evaluation of potential anti-inflammatory effects of therapeutic compounds.	The immune cells in the intestinal mucosa include natural killer T cells, innate lymphoid cells, and B lymphocytes, which are not present in peripheral blood mononuclear cells (PBMCs). These distinctions imply that PBMCs isolated from blood may demonstrate dissimilar responses to mononuclear cells in the lamina propria [257]. The selection of anticoagulants used in blood collection can impact the functionality of PBMC [266]. Monocytes derived from PBMCs depend on inflammatory mediators, such as TNF, IL-1β, or LPS, as survival factors to inhibit apoptosis [266]. The heterogeneity background among individual donors presents a common challenge in the application of PBMC-derived monocytes, resulting in considerable variations [266].

**Table 4:** Advantages and disadvantages of different animal models for IBD.

Animal Models	Advantages	Disadvantages
*2 2,4,6-trinitrobenzene sulfonic acid (TNBS)-induced colitis model*	Valuable for investigating T helper cell-dependent mucosal immune responses [278]. It is proven to be instrumental in studying various aspects of gut inflammation, including cytokine secretion patterns, oral tolerance mechanisms, cell adhesion, and immunotherapy [278]. Highlight the significance of IL-23 and IL-12 in immune response and helps to understand genetic determinants of crohn’s disease, such as NOD2. Assist in understanding the impact of host-gut microbial interactions on disease, it enables the induction of chronic transmural colitis, mimicking characteristics of Crohn’s disease, through haptenization [278].	To achieve optimal responses and to ensure reliable results requires use of a significant number of experimental mice per treatment group.
*Dextran sodium sulfate (DSS)-induced colitis model*	Valuable for exploring the involvement of innate immune mechanisms in colitis, and suitable for investigating epithelial repair mechanisms [272]. Reproducible model of colitis and allow for the induction of both acute and chronic colitis by adjusting the dosage and cycles of administration [278].	The variability between batches of DSS affects the colitis phenotype, demanding repeated administration for the induction of chronic. Lack of compliance by mice to the feeds/drinks.
*Oxazolone ((4-ethoxylmethylene-2-phenyloxazol-5-one)-induced colitis model (OC))*	Suitable for studying the contribution of the Th2-dependent immune response to intestinal inflammation [312]. At lower doses, Oxazolone can induce colitis with a mixed TH1/TH2-dependent immune response [313].	Optimizing and using a large sample size are necessary due to the high mortality of experimental animals and a dichotomous inflammatory response in this model.
*Winnie mouse model of colitis*	Exhibits symptoms closely resembling IBD.	Although this model develop inflammation similar to human IBD, the cellular and molecular pathways leading to inflammation including the role of environmental insult remain to be fully characterized.
*T cell adoptive transfer model*	Accurately replicates the pathophysiology of IBD, mainly T cell migration to the intestine. These models unveil the role of regulatory T cells in both the induction and perpetuation phases of colitis [314]. Provides flexibility in the selection of T cell types and recipient mice based on specific investigative objectives and facilitates the examination of the distinct roles of T cell subsets in intestinal homeostasis and inflammation. These models offer precise experimental control over various factors, including the timing of treatments, throughout the development of colitis.	Use of immunodeficient mice. Complicated experimental procedures are complex and time intensive. The utilization of immunodeficient mice limits the comprehensive understanding of colitis development in the context of complex factors [278]. The differences in intestinal microbiota between mice and humans may restrict their complete representation of the complexities of human IBD [303]. The application of this model involves the extraction, isolation, purification, and injection of adoptive T cells, which is complex and labour-intensive [315].

## Data Availability

Data sharing is not applicable.
